# Supplementary material for: Loss of H3K27me3 imprinting in the Sfmbt2 miRNA cluster causes enlargement of cloned mouse placentas
Source: Nat Commun. 2020 May 1;11:2150. doi: 10.1038/s41467-020-16044-8 (PMC7195362; doi:10.1038/s41467-020-16044-8)
Supplement: Supplementary file 1 — Supplementary Information [file 41467_2020_16044_MOESM1_ESM.pdf]

Supplementary Information

Loss of H3K27me3 imprinting in the Sfmbt2 miRNA cluster causes enlargement of cloned mouse placentas

Inoue et al.

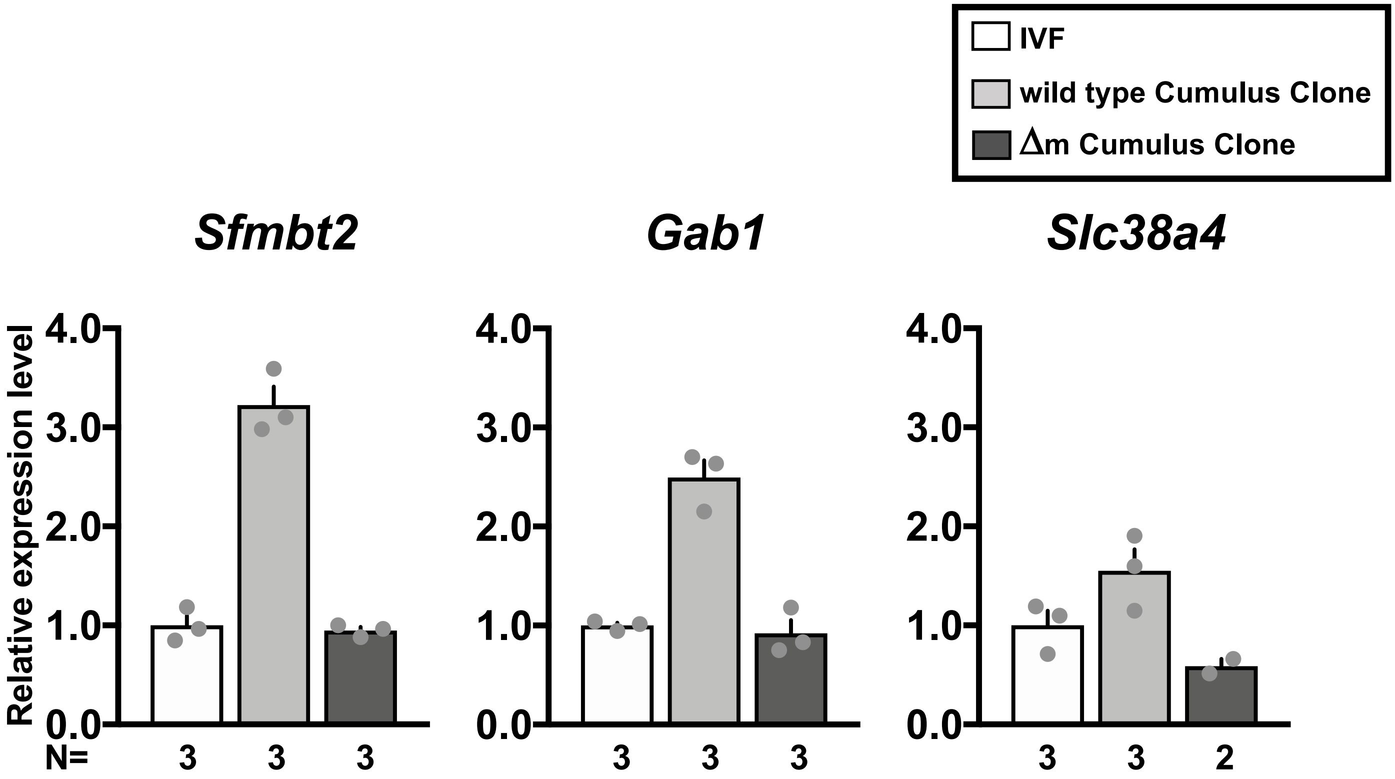

Supplementary Figure 1. Expression levels of H3K27me3-dependent imprinted genes in placentas derived from IVF, SCNT, and maternal KO SCNT embryos. The expression levels of these genes were measured using qRT–PCR. The mean value was set as 1.0 for IVF. The genes in the SCNT placentas were upregulated and those in maternal KO SCNT were corrected to the normal level. N means biological replicates. Error bars represent SEM. Source data are provided as a Source Data file.

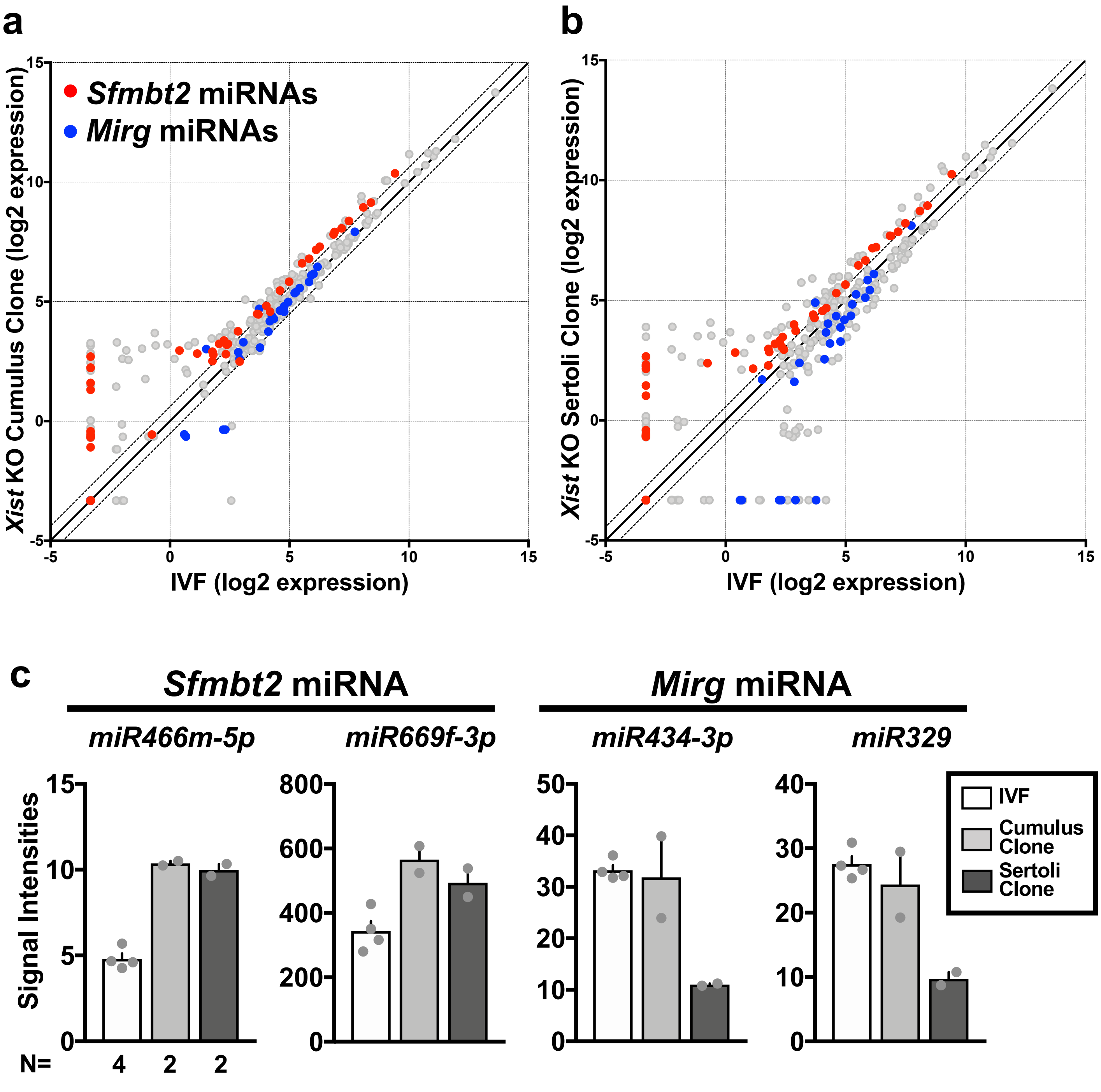

Supplementary Figure 2. Dysregulation of two miRNA clusters in *Xist* KO SCNT placentas.
(**a, b**) Scatterplot analysis between *Xist* KO cumulus (**a**) or Sertoli (**b**) SCNT and IVF placentas. Red and blue dots indicate miRNA genes in *Sfmbt2* and *Mirg* miRNA clusters, respectively. Dotted lines represent changes >1.3-fold. (**c**) Expression levels of *Sfmbt2* and *Mirg* miRNAs. N means biological replicates. Error bars represent SEM. Source data are provided as a Source Data file.

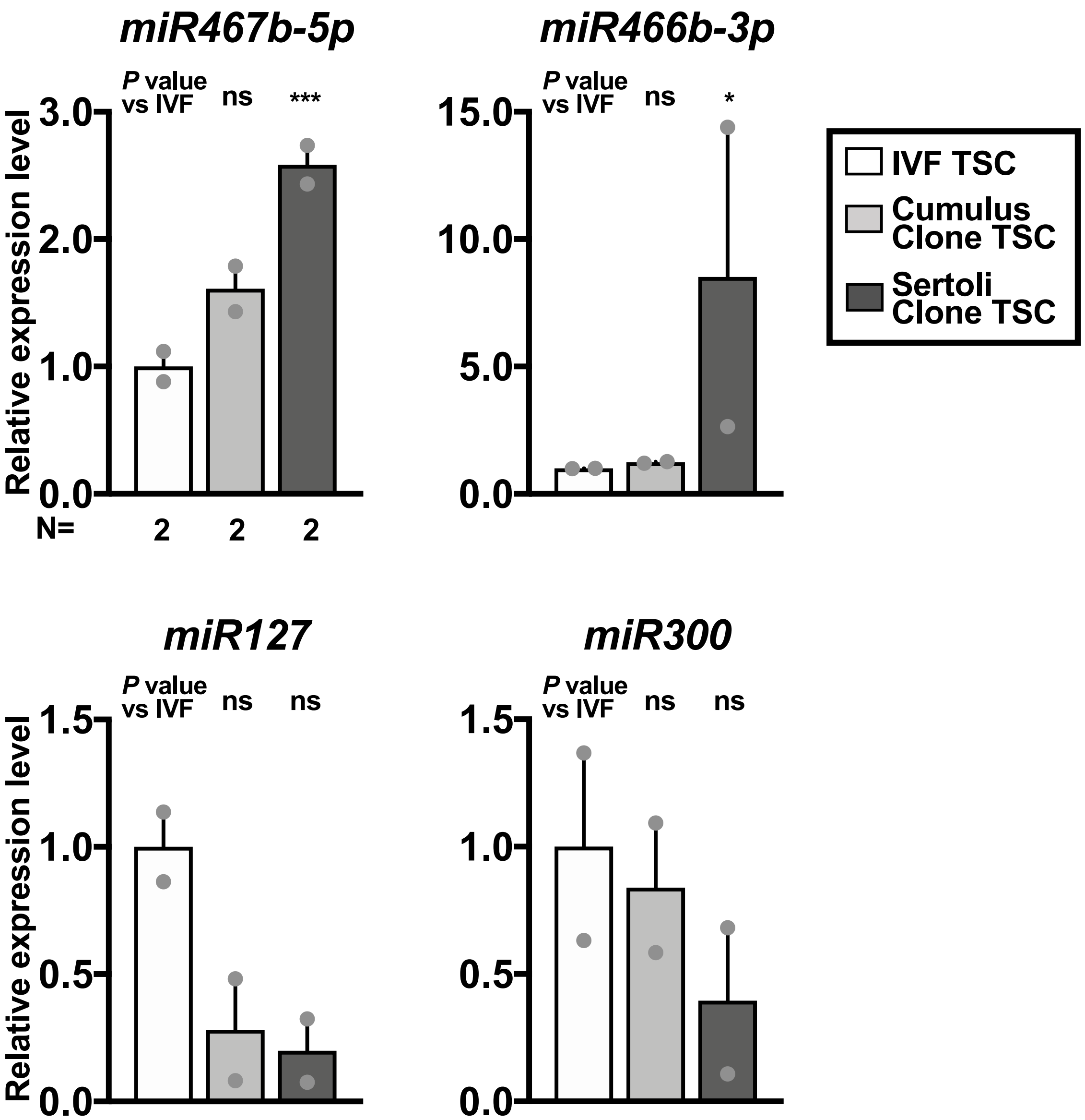

Supplementary Figure 3. Gene expression levels of two miRNA clusters in TSCs derived from IVF and SCNT embryos.

The expression levels of the *Sfmbt2* and *Mirg* miRNAs were measured using qRT-PCR. The mean value was set as 1.0 for IVF TSCs. The expression levels of the *Sfmbt2* and *Mirg* miRNAs tended to be upregulated and downregulated, respectively, in SCNT TSCs. *$P < 0.05$, ***$P < 0.005$ (Kruskal–Wallis test). N means biological replicates. Error bars represent SEM. Source data are provided as a Source Data file.

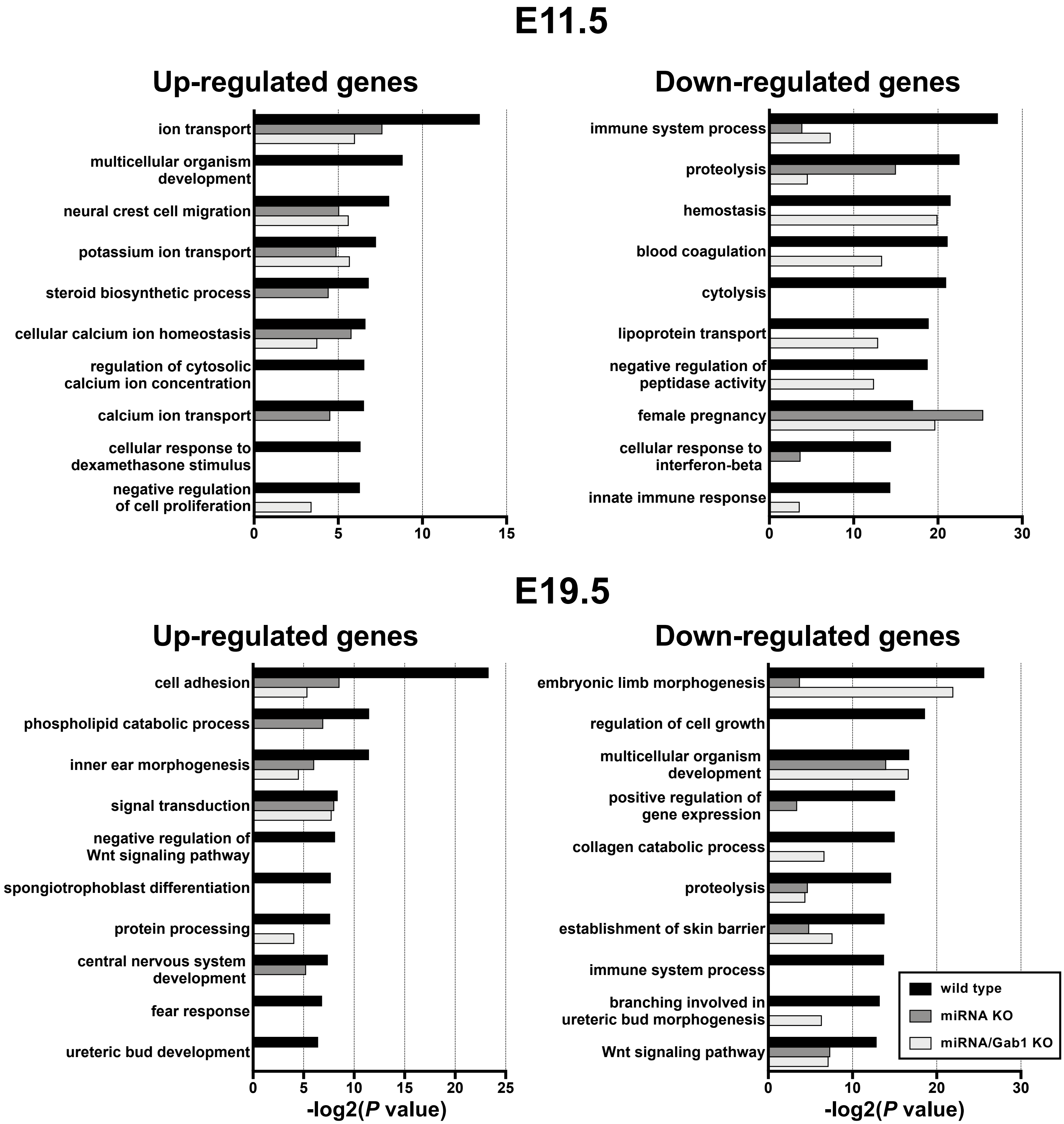

Supplementary Figure 4. Gene Ontology (GO) analysis of E11.5 and E19.5 placentas derived from SCNT fetuses with three genotypes.
Top 10 GO Biological Process terms among DEGs in the wild type SCNT and IVF placentas shown in Fig. 6b (black). The DEGs in miRNAs and double KO placentas are indicated by dark and light gray bars, respectively. The *x*-axis indicates the –log2 (*P*-value) for GO terms. Most of the *P*-values decreased for the GO terms in the miRNA or miRNA/*Gab1* KO SCNT placentas.

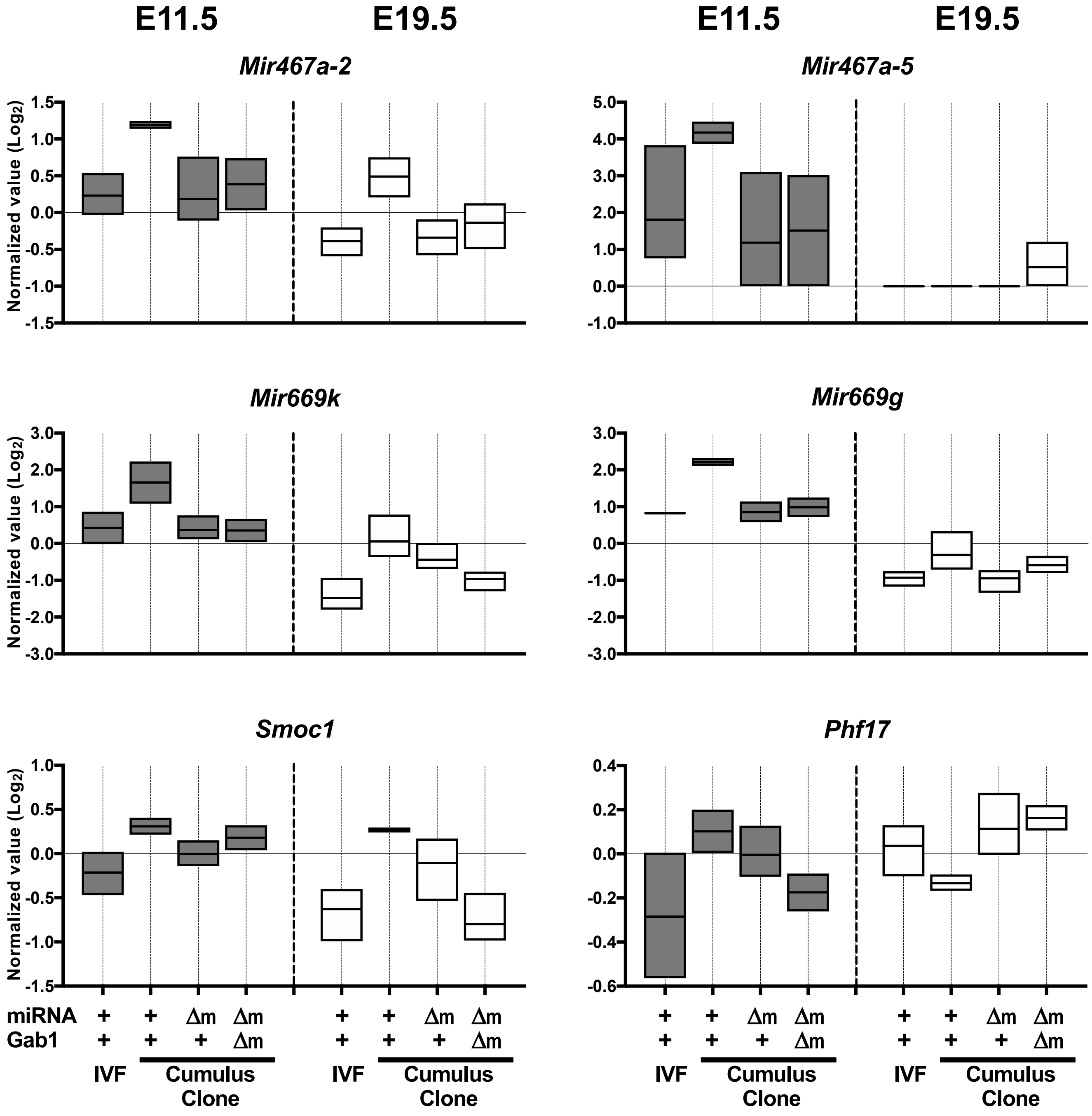

Supplementary Figure 5. Transcriptome analysis of E11.5 and E19.5 placentas derived from IVF and SCNT fetuses with three genotypes.

Expression levels of *Sfmbt2* miRNAs (*Mir467a-2*, *Mir467a-5*, *Mir669k*, and *Mir669g*) and placenta-specific imprinted genes (*Smoc1* and *Phf17*) in E11.5 and E19.5 IVF and SCNT placentas. Upper, lower and center lines indicate minimum, maximum and mean values. Source data are provided as a Source Data file.

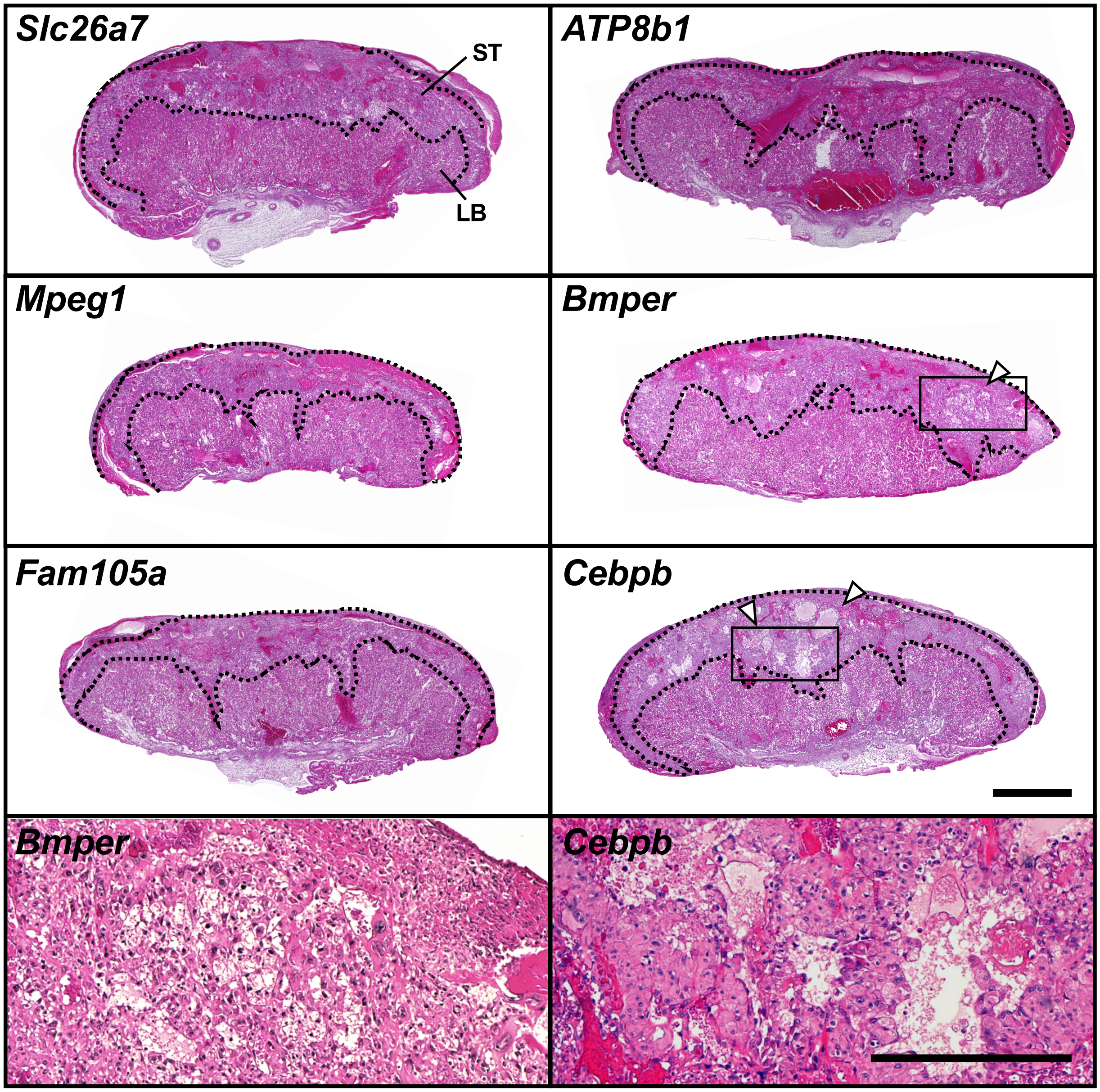

Supplementary Figure 6. E19.5 placental histology of predicted target gene KO mice. Hematoxylin and eosin-stained tissue sections of E19.5 placentas derived from six predicted target gene KO mice. Dotted lines indicate the boundaries between decidua, ST, and LB layers. In *Bmper and Cebpb* KO placentas, increases in the numbers of glycogen cells were observed (arrowheads). Distortions of ST–LB boundaries were observed in all KO placentas. ST, spongiotrophoblast layer; LB, labyrinthine layer. Scale bar, 1 mm. The bottom two figures show magnified images of ST layers (framed by squares) in *Bmper* and *Cebpb* KO placentas. Scale bar, 0.5 mm.

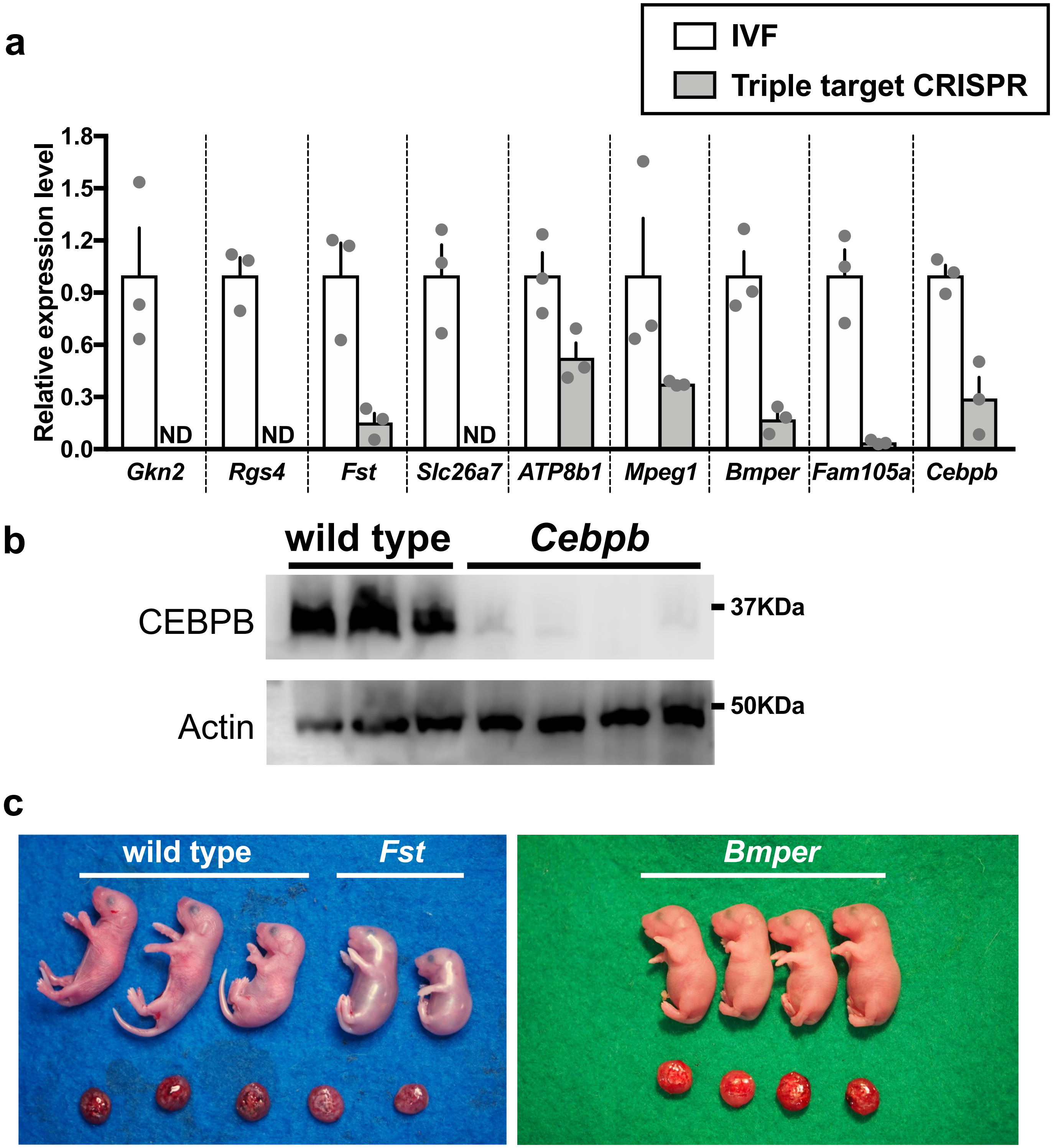

Supplementary figure 7. Expression levels of target genes in triple target CRISPR and neonates of *Fst* and *Bmper* KO. (**a**) Expression levels of target genes in the placentas of triple target CRISPR animals, as measured using qRT–PCR. The mean values of IVF placentas were set as 1.0. Three biological replicates and two technical replicates were used in each group. The expression levels of all target genes were significantly decreased compared with those of IVF placentas ($P < 0.05$, nonparametric *t*-test). Error bars represent SEM. (**b**) Western blot analysis of *Cebpb* target CRSPR placentas. Protein signals were not detected in the placentas of *Cebpb* targeted animals. CEBPB and Actin indicate the antibodies that were used for immunoblotting. (**c**) Neonates of *Fst* and *Bmper* KO. All neonates died shortly after birth[39,40] and *Fst* KO neonates exhibited a shiny and taut skin, as described previously[39]. Source data of Supplementary Figures 7a and b are provided as a Source Data file.
