# Supplementary material for: Loss of H3K27me3 imprinting in the Sfmbt2 miRNA cluster causes enlargement of cloned mouse placentas
Source: Nat Commun. 2020 May 1;11:2150. doi: 10.1038/s41467-020-16044-8 (PMC7195362; doi:10.1038/s41467-020-16044-8)
Supplement: Supplementary file 3 — Reporting Summary [file 41467_2020_16044_MOESM3_ESM.pdf]

nature research

Corresponding author(s): Kimiko Inoue, Atsuo Ogura

Last updated by author(s): Mar 5, 2020

Reporting Summary

Nature Research wishes to improve the reproducibility of the work that we publish. This form provides structure for consistency and transparency in reporting. For further information on Nature Research policies, see Authors & Referees and the Editorial Policy Checklist.

Statistics

For all statistical analyses, confirm that the following items are present in the figure legend, table legend, main text, or Methods section.

n/a	Confirmed	
☐	☒	The exact sample size (*n*) for each experimental group/condition, given as a discrete number and unit of measurement
☐	☒	A statement on whether measurements were taken from distinct samples or whether the same sample was measured repeatedly
☐	☒	The statistical test(s) used AND whether they are one- or two-sided<br>*Only common tests should be described solely by name; describe more complex techniques in the Methods section.*
☒	☐	A description of all covariates tested
☐	☒	A description of any assumptions or corrections, such as tests of normality and adjustment for multiple comparisons
☐	☒	A full description of the statistical parameters including central tendency (e.g. means) or other basic estimates (e.g. regression coefficient) AND variation (e.g. standard deviation) or associated estimates of uncertainty (e.g. confidence intervals)
☐	☒	For null hypothesis testing, the test statistic (e.g. *F*, *t*, *r*) with confidence intervals, effect sizes, degrees of freedom and *P* value noted<br>*Give P values as exact values whenever suitable.*
☒	☐	For Bayesian analysis, information on the choice of priors and Markov chain Monte Carlo settings
☒	☐	For hierarchical and complex designs, identification of the appropriate level for tests and full reporting of outcomes
☒	☐	Estimates of effect sizes (e.g. Cohen's *d*, Pearson's *r*), indicating how they were calculated

Our web collection on statistics for biologists contains articles on many of the points above.

Software and code

Policy information about availability of computer code

Data collection

analysis of miRNA data: GeneSpring GX (Agilent);
alignment and analysis of transcriptome: Strand NGS (Agilent);
Gene Ontology analysis: DAVID Bioinformatics Resources (https://david-d.ncifcrf.gov);
analysis of miRNA target genes: TargetScan (http://www.targetscan.org/mmu_72/) and miRNA.org (http://www.microrna.org/microrna/home.do);
statistical analysis: Prism (GraphPad)

Data analysis

The selection of DEGs from the miRNA microarray data was performed by Gene Spring GX (version 12.5). Statistically significant DEGs between IVF (n = 4) and cumulus-derived (wild-type, n = 3; Xist KO, n = 2) or Sertoli-derived (wild-type, n = 3; Xist KO, n = 2) cloned placentas were extracted by moderated t-test prior to Benjamini and Hochberg FDR procedure and >1.3-fold change.

The transcriptome analysis reported in Figures 6 and 7, and Supplementary Figures 4 and 5 was performed by Strand NGS. Genes with a low expression level and raw values that were <20 in all samples were excluded from further analysis. Passed raw values were normalized via DE-seq and baseline treatment was performed using the default setting of the software. Genes that were differentially expressed by more than 1.8-fold between IVF and each SCNT placenta groups were determined as being DEGs. The number of analyzed IVF, wild-type, miRNA, and miRNA/Gab1 KO placentas at E11.5 was 3, 2, 3, and 2, respectively. At E19.5, three placentas were analyzed in each genotype.

For the statistical analysis of placental weight shown in Figure 1a and 5a, the Kruskal–Wallis test followed by Dunn's multiple comparison test were used. In Figure 5b, two-way ANOVA followed by Dunnet's multiple comparison test were used. The number of measured IVF, wild-type, miRNA KO, and miRNA/Gab1 KO placentas was 7, 4, 3, and 4, respectively.

In Supplementaly Figure 3, the Kruskal–Wallis test followed by Dunn's multiple comparison test were used. In Supplementary Figure 7a, t-test was performed in each gene.

All error bars within graphs indicate the S.E.M. and the horizontal bars indicate the mean values. In all statistical analyses, significance was set at P < 0.05.

For manuscripts utilizing custom algorithms or software that are central to the research but not yet described in published literature, software must be made available to editors/reviewers. We strongly encourage code deposition in a community repository (e.g. GitHub). See the Nature Research guidelines for submitting code & software for further information.

Data

Policy information about availability of data

All manuscripts must include a data availability statement. This statement should provide the following information, where applicable:

- Accession codes, unique identifiers, or web links for publicly available datasets
- A list of figures that have associated raw data
- A description of any restrictions on data availability

All expression profile data reported in this paper were deposited into NCBI GEO Dataset: GSE129940 (miRNA microarray and RNA-seq).
for miRNA analysis: Figures 2 and 3, and Supplementary figure 2;
for transcriptome analysis: Figures 6 and 7, and Supplementary figures 4 and 5.

Field-specific reporting

Please select the one below that is the best fit for your research. If you are not sure, read the appropriate sections before making your selection.

☒ Life sciences ☐ Behavioural & social sciences ☐ Ecological, evolutionary & environmental sciences

For a reference copy of the document with all sections, see nature.com/documents/nr-reporting-summary-flat.pdf

Life sciences study design

All studies must disclose on these points even when the disclosure is negative.

Sample size	Two to four biological replicates in miRNA, transcriptome and RT-PCR analysis were chosen according to common standard for these type of assays.
Data exclusions	In miRNA microarray experiment, two samples (one cumulus Xist KO- and one Sertoli Xist KO-derived placentas) were excluded, because their hybridization qualities were low and their box plots were out of normal range.
Replication	for microarray of miRNA: The number of analyzed IVF, cumulus-, Sertoli-, cumulus Xist KO- and Sertoli Xist KO-derived cloned placentas was 4, 3, 3, 2 and 2, respectively.<br>for transcriptome analysis: The number of analyzed IVF, wild-type, miRNA, and miRNA/Gab1 KO placentas at E11.5 was 3, 2, 3, and 2, respectively. At E19.5, three placentas were analyzed in each genotype.<br>for qRT-PCR analysis:Three biological and two technical replicates were used in IVF and wild-type SCNT placentas in Supplementary figure 1.<br>for maternal KO SCNT placentas, three biological replicates in Sfmbt2 and Gab1 KO, and two biological replicates in Slc38a4 KO were used for measurement, respectively. Two technical replicates were used in these samples. Two biological and two technical replicates were used in each group in Supplementary figure 3. Three biological and two technical replicates were used in Supplementary figure 7a.<br>for analysis of placental weights: The numbers are 37, 6, 6, 9 and 3 for IVF, wild-type SCNT, Sfmbt2 KO, Gab1 KO and Slc38a4 KO SCNT shown in Figure 1a, and 37, 6, 5 and 9 for IVF, wild-type SCNT, miRNA KO and miRNA/Gab1 KO SCNT in Figure 5a.<br>for measurement of placental area: The number of IVF, wild-type, miRNA KO, and miRNA/Gab1 KO placentas was 7, 4, 3, and 4, respectively.
Randomization	All animals used in this study were used during eight- to 12-week-old in the order of age. Apparent non-healthy animals were excluded from pools.
Blinding	No blinding was applicable in this study.

Reporting for specific materials, systems and methods

We require information from authors about some types of materials, experimental systems and methods used in many studies. Here, indicate whether each material, system or method listed is relevant to your study. If you are not sure if a list item applies to your research, read the appropriate section before selecting a response.

Materials & experimental systems

n/a	Involved in the study	
☐	☒	Antibodies
☒	☐	Eukaryotic cell lines
☒	☐	Palaeontology
☐	☒	Animals and other organisms
☒	☐	Human research participants
☒	☐	Clinical data

Methods

n/a	Involved in the study	
☒	☐	ChIP-seq
☒	☐	Flow cytometry
☒	☐	MRI-based neuroimaging

Antibodies

Antibodies used	anti-DIG AP conjugate (Sigma-Aldrich, catalog number: 11093274910)<br>anti-CEBPB (Abcam, catalog number: ab32358)<br>anti-Actin (Santa Cruz Biotechnology, catalog number: sc-1616)
Validation	commercially available HRP conjugated 2nd antibody (Millopore)

Animals and other organisms

Policy information about studies involving animals; ARRIVE guidelines recommended for reporting animal research

Laboratory animals	Species: Mus musculus domesticus<br>Strain: oocyte collection for nuclear transfer, eight- to 10-week-old (C57BL/6 (B6) x DBA/2) F1 (BDF1) female mice;<br>nuclear donor, BDF1 wild-type and KO mice;<br>embryo transfer recipient, eight- to 12-week-old ICR female mice;<br>CRISPR/Cas9 mice, BDF1 x B6 or B6 x B6 in founder generation. The founder mice obtained were crossed with B6 wild-type mice for two or three generations and used in further experiments.;<br>triple target CRISPR: BDF1 x B6 mice.
Wild animals	No wild animals were used in this study.
Field-collected samples	No field-collected samples were used in this study.
Ethics oversight	The care and use of animals in this study was performed with the guidance for use and maintenance of experimental animals from Japanese Ministry of Environment. All animal experiments in this study were approved by the Institutional Animal Care and Use Committee of RIKEN Tsukuba Branch.

Note that full information on the approval of the study protocol must also be provided in the manuscript.
