# Supplementary material for: Loss of H3K27me3 imprinting in the Sfmbt2 miRNA cluster causes enlargement of cloned mouse placentas
Source: Nat Commun. 2020 May 1;11:2150. doi: 10.1038/s41467-020-16044-8 (PMC7195362; doi:10.1038/s41467-020-16044-8)
Supplement: Supplementary file 4 — Description of Additional Supplementary Files [file 41467_2020_16044_MOESM4_ESM.pdf]

Description of Additional Supplementary Files

File Name: Supplementary Data 1
Description: List of differentially expressed miRNAs between IVF and cumulus- or Sertoli-cloned placentas at E11.5.

File Name: Supplementary Data 2
Description: List of upregulated genes in wild-type, miRNA KO, and miRNA/Gab1 KO SCNT placentas at E11.5.

File Name: Supplementary Data 3
Description: List of downregulated genes in wild-type, miRNA KO, and miRNA/Gab1 KO SCNT placentas at E11.5.

File Name: Supplementary Data 4
Description: List of upregulated genes in wild-type, miRNA KO, and miRNA/Gab1 KO SCNT placentas at E19.5.

File Name: Supplementary Data 5
Description: List of downregulated genes in wild-type, miRNA KO, and miRNA/Gab1 KO SCNT placentas at E19.5.

File Name: Supplementary Data 6
Description: Biased chromosomal location of up- and downregulated genes in E11.5 wild-type SCNT placentas.

File Name: Supplementary Data 7
Description: The genes, number, and target sequences of triple-targeted CRISPR placentas.

File Name: Supplementary Data 8
Description: Sequences of the qRT–PCR primer sets.
